# Chemical and genetic validation of dihydrofolate reductase–thymidylate synthase as a drug target in African trypanosomes

**DOI:** 10.1111/j.1365-2958.2008.06305.x

**Published:** 2008-06-16

**Authors:** Natasha Sienkiewicz, Szymon Jarosławski, Susan Wyllie, Alan H Fairlamb

**Affiliations:** Division of Biological Chemistry & Drug Discovery, College of Life Sciences, University of DundeeDundee, UK

## Abstract

The phenotypes of single- (SKO) and double-knockout (DKO) lines of dihydrofolate reductase–thymidylate synthase (DHFR–TS) of bloodstream *Trypanosoma brucei* were evaluated *in vitro* and *in vivo*. Growth of SKO *in vitro* is identical to wild-type (WT) cells, whereas DKO has an absolute requirement for thymidine. Removal of thymidine from the medium triggers growth arrest in S phase, associated with gross morphological changes, followed by cell death after 60 h. DKO is unable to infect mice, whereas the virulence of SKO is similar to WT. Normal growth and virulence could be restored by transfection of DKO with *T. brucei* DHFR–TS, but not with *Escherichia coli* TS. As pteridine reductase (PTR1) levels are unchanged in SKO and DKO cells, PTR1 is not able to compensate for loss of DHFR activity. Drugs such as raltitrexed or methotrexate with structural similarity to folic acid are up to 300-fold more potent inhibitors of WT cultured in a novel low-folate medium, unlike hydrophobic antifols such as trimetrexate or pyrimethamine. DKO trypanosomes show reduced sensitivity to these inhibitors ranging from twofold for trimetrexate to >10 000-fold for raltitrexed. These data demonstrate that DHFR–TS is essential for parasite survival and represents a promising target for drug discovery.

## Introduction

The post-genomic era offers unparalleled opportunities for the identification, characterization and validation of novel molecular targets for drug discovery in order to replace the currently unsatisfactory therapies for human African trypanosomiasis. The initial selection of potential targets from the ‘druggable genome’ is of crucial importance and known targets of current drugs in clinical use for other diseases are a useful starting point. Folic acid metabolism is one such area with clinical precedents in bacterial or protozoan infections and certain human malignancies (Blaney *et al.*, 1984; McGuire, 2003; Gangjee *et al.*, 2007).

Folate and its derivatives are essential cofactors in one-carbon metabolism that are required for the biosynthesis of purines, thymidylate, serine and methionine in a wide variety of organisms. Most bacteria, some protozoa (e.g. malaria), fungi and plants synthesize folates *de novo* using a pterin (from GTP), *p*-aminobenzoate (via the chorismate pathway) and glutamate. In contrast, trypanosomatids and their mammalian hosts lack a *de novo* folate-synthesis pathway and thus require exogenous folate for these biosynthetic functions.

**Fig. 1:**
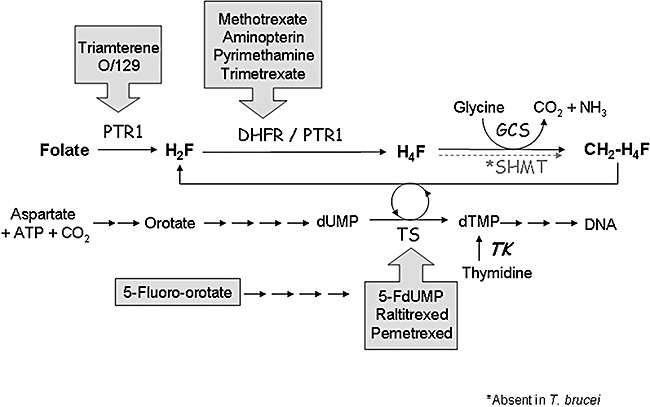
Pathway of thymidylate synthesis and primary site of action of inhibitors. SHMT, serine hydroxymethyltransferase; DHFR, dihydrofolate reductase; TS, thymidylate synthase; O/129, 2,4-diamino-6,7-diispropylpteridine; PTR1, pteridine reductase 1; TK, thymidine kinase; H_2_F, dihydrofolate; H_4_F, tetrahydrofolate; CH_2_-CH_4_F, 5, 10-methylene-tetrahydrofolate; FdUMP, 5-fluorodeoxyuridylate; GCS, glycine cleavage system.

Trypanosomatids have lost the ability to synthesize purines and therefore salvage them from their environment, yet have retained the complete biosynthetic pathway to pyrimidines necessary for nucleic acid synthesis (Fig. 1). A key step in DNA synthesis is formation of thymidylate (dTMP) catalysed by thymidylate synthase (TS; EC 2.1.1.45) involving the reductive methylation of deoxyuridylate (dUMP) by 5, 10-methylene-tetrahydrofolate (CH_2_-H_4_F). The other product of this reaction, dihydrofolate (H_2_F), is converted into tetrahydrofolate (H_4_F) by dihydrofolate reductase (DHFR; EC 1.5.1.3). Finally CH_2_-H_4_F is regenerated from H_4_F via either serine hydroxymethyltransferase (EC 2.1.2.1) or the glycine cleavage system to complete the reaction cycle. In trypanosomatids and other parasites, DHFR and TS are fused to form a bifunctional protein, unlike their mammalian hosts.

Selective inhibition of DHFR or TS in prokaryotic and eukaryotic cells results in ‘thymine-less death’ by necrosis or apoptosis as a consequence of thymidine starvation (Ahmad *et al.*, 1998; Van Triest *et al.*, 2000). However, DHFR–TS has not been successfully exploited so far for treatment of either trypanosomal or leishmanial infections (Nare *et al.*, 1997a;Linares *et al.*, 2006). Although the reason for this is not at all clear for African trypanosomes, in *Leishmania* spp., pteridine reductase (PTR1; EC 1.5.1.33) may serve to modulate or by-pass inhibition of DHFR by classical inhibitors such as methotrexate (Nare *et al.*, 1997a). Gene knockout studies in avirulent lines of *Leishmania major* have shown that null mutants of *DHFR–TS* can be readily generated when supplemented with thymidine (Cruz and Beverley, 1990; Cruz *et al.*, 1991). In contrast, virulent *Leishmania* show plasticity in chromosome number in order to maintain at least one copy of *DHFR–TS* (Cruz *et al.*, 1993). Thus, DHFR–TS may have another unidentified function in virulent lines in addition to *de novo* synthesis of thymidine in avirulent lines. Whether this requires DHFR, TS or both proteins is not clear. Neither is it clear whether endogenous PTR1 activity is sufficient to replace DHFR in the thymidylate cycle.

Very little is known about folate metabolism in African trypanosomes. Comparative genomics indicates that *Trypanosoma brucei* lacks a number of genes in folate-dependent pathways that are present in *L. major* indicating that extrapolation from *L. major* to *T. brucei* may not be straightforward (Berriman *et al.*, 2005). Moreover, from a drug discovery perspective, although *T. brucei* possess both DHFR–TS and PTR1 (Gamarro *et al.*, 1995; Dawson *et al.*, 2006), these enzymes show significant differences in structure and/or sensitivity to inhibitors to their *Leishmania* counterparts (Meek *et al.*, 1985; Hardy *et al.*, 1997; Luba *et al.*, 1998; Chowdhury *et al.*, 1999; Gourley *et al.*, 1999; 2001). In this study we use genetic and chemical methods to examine the roles of DHFR–TS in *T. brucei* and assess its potential as a drug target. We also examine the sensitivity of *T. brucei* bloodstream forms to known DHFR–TS inhibitors in a novel culture medium containing physiological levels of folate to assess the robustness of the currently accepted standard method for whole cell phenotypic screening of antifols (Raz *et al.*, 1997).

## Results

### Generation of knockout mutants

**Fig. 2:**
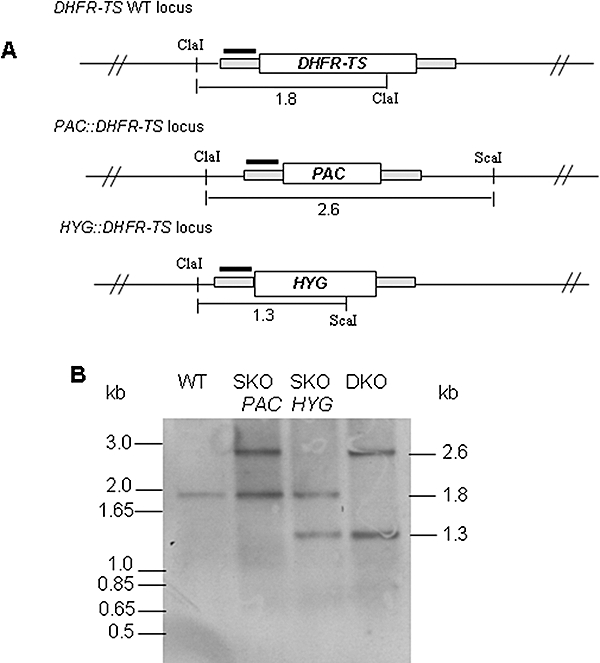
Genotypic analysis of WT, SKO and DKO cells. A. Structure of the DHFR–TS locus and predicted replacements. The black bars represent the 5′-UTR region upstream of the open reading frames of *DHFR–TS*, *PAC* and *HYG* used as a probe in Southern blot analysis. Only relevant restriction enzyme sites with expected fragment sizes are shown. B. Southern analysis of DNA digested with ClaI and ScaI from WT, SKO (containing PAC and HYG respectively) and DKO cells. DNA size markers are on the left-hand side of blots and the estimated size of detected fragments on the right.

To assess the essentiality of *DHFR–TS*, bloodstream-form trypanosomes were transfected with linear constructs containing the drug resistance genes, puromycin *N*-acetyl transferase (*PAC*) or hygromycin phosphotransferase (*HYG*) flanked by the 3′- and 5′-untranslated regions (3′-UTR and 5′-UTR respectively) of *DHFR–TS.* Stable drug-resistant lines were obtained after selection with either puromycin or hygromycin in HMI9 medium. This culture medium contains 160 μM thymidine which serves as a nutritional by-pass for loss of DHFR–TS activity. The resulting single-knockout (SKO) line containing *PAC* was then transfected with the *HYG* knockout construct and selected for resistance to puromycin and hygromycin to obtain a double-knockout (DKO) line. A Southern blot of a restriction enzyme digest of genomic DNA from wild type (WT) (*DHFR–TS*/*DHFR–TS*), SKO (*DHFR–TS*/*dhfr–ts*::*PAC*) and DKO (*dhfr–ts*::*PAC*/*dhfr–ts*::*HYG*) confirmed that both drug selectable markers had correctly integrated into the S427 genome via homologous recombination at the *DHFR–TS* locus (Fig. 2).

### Effects of folate or thymidine on growth

**Fig. 3:**
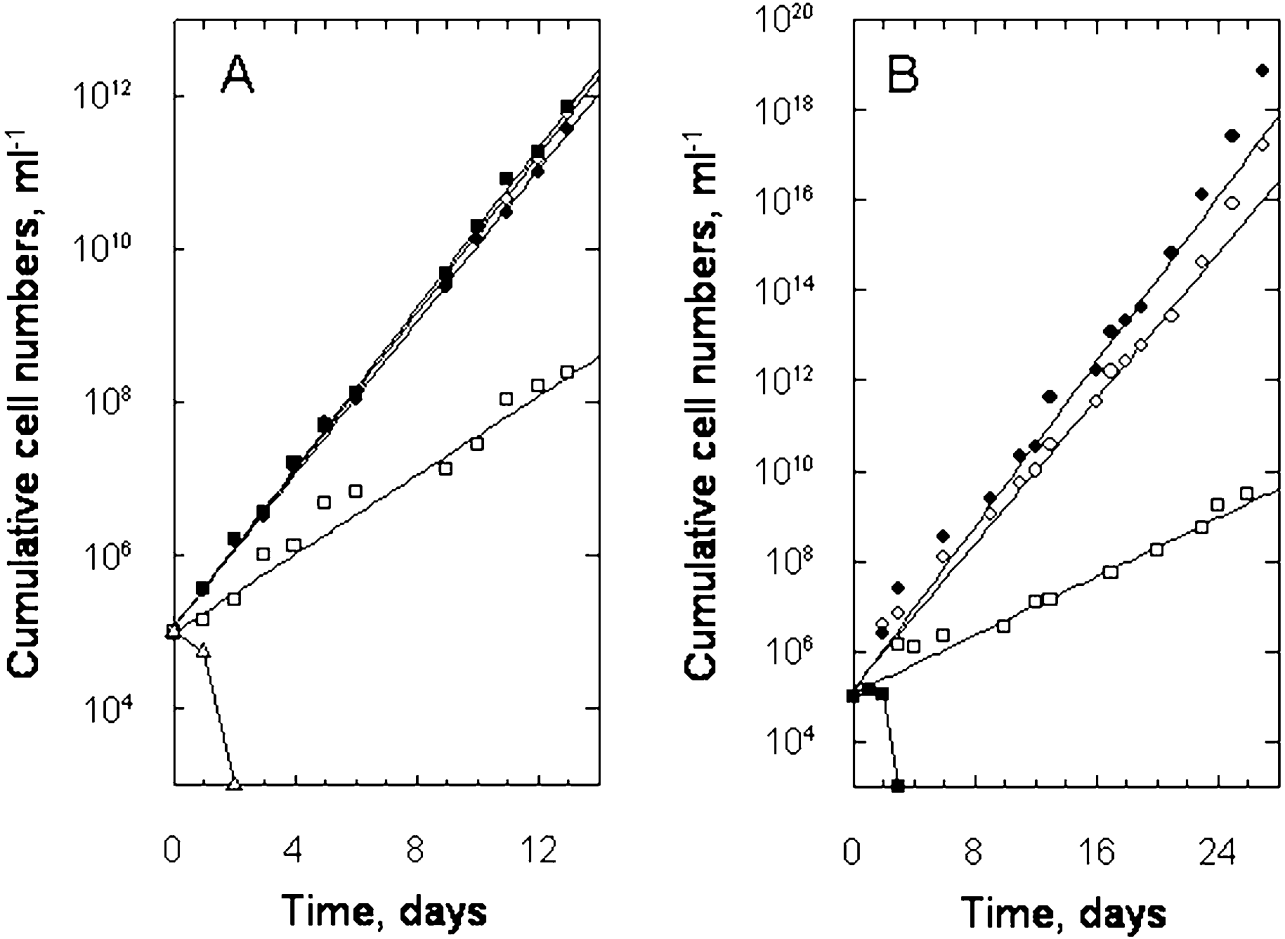
Growth characteristics of the *T. brucei* WT, SKO and DKO cells under various conditions. A. Growth in HMI9 medium in the presence/absence of drug selection. WT, open circles; SKO plus puromycin, closed circles; DKO plus puromycin and hygromycin, open squares; and DKO without drugs, closed squares. Open triangles show the growth of DKO without drugs in HMI9 medium lacking the thymidine component. B. Growth in folate-deficient media (FDM) containing 160 μM thymidine. WT, open circles; SKO plus puromycin, closed circles; DKO plus puromycin and hygromycin, open squares; and DKO without thymidine, closed squares. Data are the means of triplicate cultures, where the standard deviations are 5% or less of the value. Symbols on the *x*-axis indicate that cell densities are below the limits of detection (i.e. < 10^3^ ml^−1^). The lines represent the best fits to the equation describing exponential growth as described in *Experimental procedures*.

Cell lines were continuously passaged in various media to determine growth phenotypes. In HMI9 medium (Fig. 3A), WT and SKO cells grew at similar rates with doubling times of 14.0 ± 0.1 and 14.5 ± 0.1 h respectively. The DKO cell line grew at a decreased rate in the presence of puromycin plus hygromycin with a generation time of 28.0 ± 1.3 h. However, in the absence of drug selection, growth of DKO parasites was similar to that of the other cell lines (13.8 ± 0.1 h). Folate-deficient medium (FDM) was developed to approximate the physiological levels found in mouse (95 nM) (Clarke *et al.*, 2000) and human plasma (16–66 nM) (Hannon-Fletcher *et al.*, 2004; Lin *et al.*, 2004). Similar generation times were obtained for WT and SKO trypanosomes in FDM that are marginally slower compared with HMI9 (18.0 ± 0.4 and 15.8 ± 0.4 h respectively) (Fig. 3B). DKO grew considerably slower (44 ± 2 h) in this medium in the absence of drug selection. Removal of thymidine from either medium did not affect growth of WT or SKO cells, whereas the DKO failed to grow and died by 72 h (Fig. 3).

**Fig. 4:**
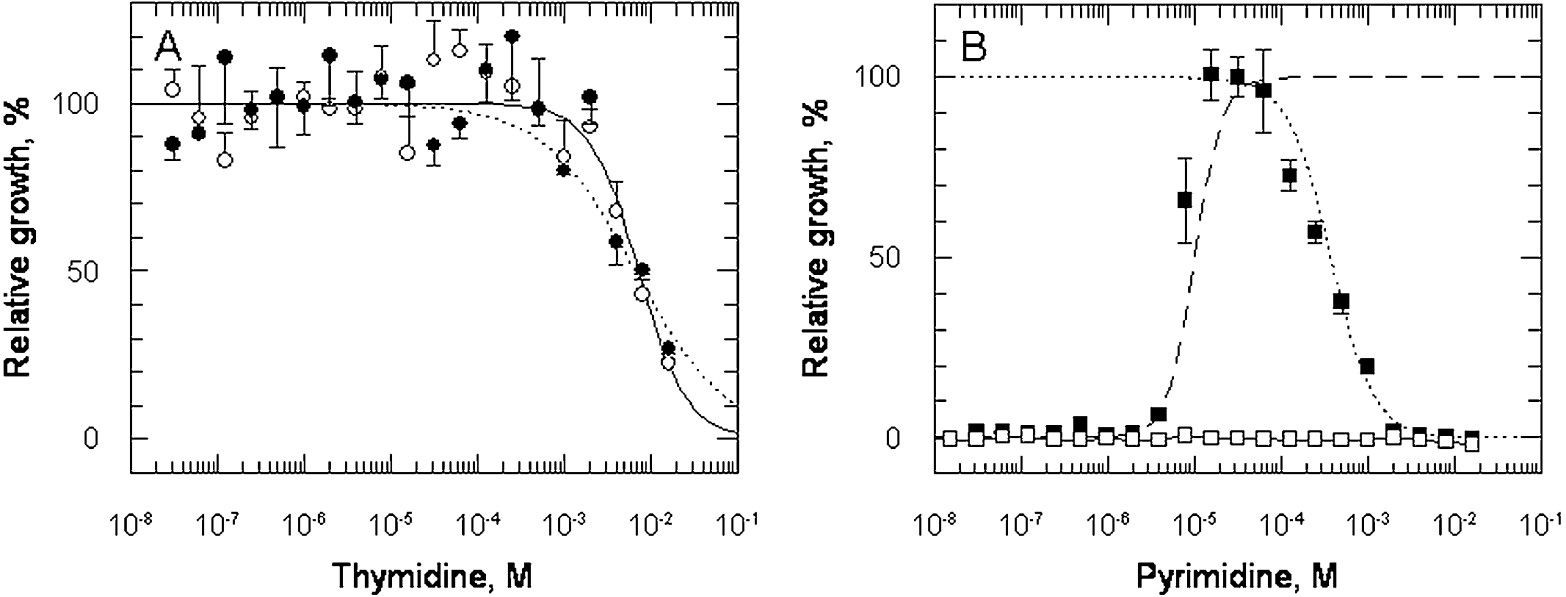
Effect of pyrimidines on growth. Cells were grown in HMI9 medium supplemented with varying amounts of pyrimidines for 72 h and cell density was determined using Alamar Blue as described in *Experimental procedures*. A. WT plus thymidine, open circles; SKO plus thymidine, closed circles. B. DKO plus thymidine, closed squares; DKO plus thymine, open squares (a complete lack of growth stimulation was also observed with uracil or uridine). The dashed line is the best fit for growth stimulation and the dotted line is the best fit for growth inhibition.

To determine the effects of thymidine concentration on growth, WT, SKO and DKO cell lines were cultured in thymidine-free HMI9 supplemented with varying amounts of thymidine or other pyrimidines (Fig. 4). Thymidine had no effect on growth of WT or SKO cells up to 1 mM, but was toxic at higher concentrations giving similar EC_50_ values of 7.3 ± 0.6 and 6.7 ± 0.7 mM for WT and SKO respectively (Fig. 4A). In contrast, DKO cells display a narrow window of growth, optimal at 16–64 μM, with essentially no growth with < 2 μM or > 2 mM thymidine (Fig. 4B). Higher concentrations were toxic to DKO cells with an EC_50_ of 380 ± 45 μM. Other pyrimidines (thymine, uracil or uridine) were unable to replace thymidine (Fig. 4B). To determine at which point ‘thymidine-less death’ was irreversible in the DKO line, cells were incubated in thymidine-free HMI9 and then transferred back into thymidine-replete HMI9 medium at intervals. Samples were cultured for up to 14 days and examined daily for the emergence of live, motile parasites. Under these conditions, viable parasites were recovered from cultures depleted of thymidine for up to 54 h, but not at 60 h or thereafter (data not shown).

### DNA content and morphology of WT and DKO cells

**Fig. 5:**
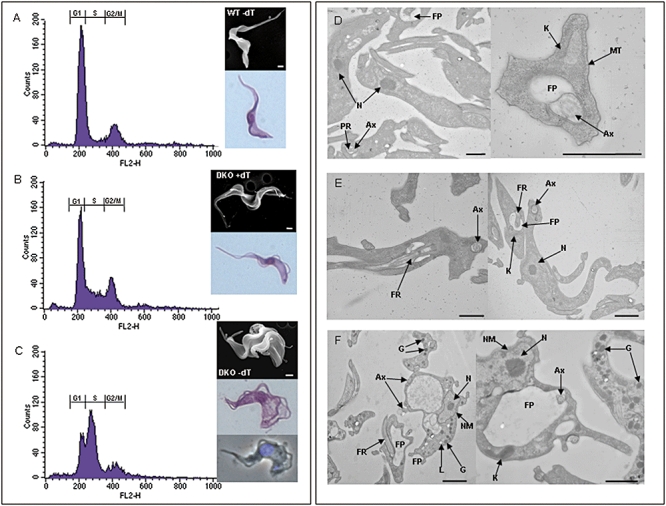
Phenotypic analysis WT and DKO cells grown in the presence or absence of thymidine. Cells were grown in either the absence or presence of thymidine (dT) for 24 h and prepared for FACS, SEM, Giemsa, DAPI and TEM analysis. The histograms from the FACS analysis are presented in (A) WT minus thymidine, (B) DKO plus thymidine, (C) DKO minus thymidine with SEM, Giemsa and DAPI images of the respective cells in the right-hand panels. TEM showing the morphology of the WT (D), DKO plus thymidine (E) and DKO minus thymidine (F) are depicted. Bars represent 1 μm. N, nucleus; NM, nuclear membrane; FR, flagella rod; FP, flagella pocket; Ax, axonemes; G, glycosome; L, lysosome; PR, paraxial rod; MT, microtubules.

Prokaryotic and eukaryotic organisms that are auxotrophic for thymidine undergo cell death by necrosis or apoptosis in response to thymidine starvation. To establish whether the DKO cells undergo a similar response, the DNA content was analysed using a fluorescence-activated cell sorter (FACS) as illustrated in Fig. 5A–C. For the WT cell population, 72% were in G1 phase, with 7% in S (DNA synthesis) phase and 16% in G2/M phase (Fig. 5A). In contrast, DKO cells grown in thymidine-deficient medium were mainly arrested in S phase (58%), with proportionally less in G1 or G2/M (Fig. 5C). In contrast, DKO cells grown with thymidine showed a similar profile to WT cells (Fig. 5B). Thus, thymidine starvation results in cells failing to complete DNA synthesis from 2N (G1) to 4N (G2/M) with arrest within S phase.

The morphology of DKO cells grown in the absence of thymidine for 24 h was examined by scanning electron, light and fluorescence microscopy. In contrast to the long slender morphology of WT or DKO trypanosomes grown in the presence of thymidine (Fig. 5A and B), most thymidine-depleted DKO trypanosomes appeared shorter and fatter with loss of their free flagellum. In some respects these are morphologically similar to the non-dividing stumpy forms seen in relapsing infections which are metabolically pre-adapted for growth in the tsetse fly vector midgut (Fairlamb and Opperdoes, 1986). However, unlike stumpy forms, some had lost their flagellum or possessed two flagella (Fig. 5C). A large vacuole at the posterior end of the parasite was also evident, which, by transmission electron microscopy, appeared to be a grossly enlarged flagellar pocket (Fig. 5F) compared with normal WT and DKO sections (Fig. 5D and E respectively). The nucleus is also enlarged and is associated with a noticeable increase in the number of glycosomes and lysosomes present throughout the sections compared with normal WT and DKO cells. No evidence for death by an apoptotic-like event was detected, either by staining live cells with Annexin V-FITC (Wyllie and Fairlamb, 2006) or by analysis of DNA for characteristic fragmentation patterns (Ioannou and Chen, 1996) (data not shown).

### Effect of antifolate and PTR1 inhibitors

A range of DHFR–TS and PTR1 inhibitors were tested against WT and DKO trypanosomes to assess specificity against their predicted targets (see Fig. 1). As folate levels in the culture medium can also modulate drug sensitivity, EC_50_ values for growth inhibition were determined in high- (HMI9; 8 μM) and low- (FDM; ∼30 nM) folate media (Table 1). In high-folate media, none of the nine inhibitors tested were particularly effective against WT cells (EC_50_ values > 1 μM), whereas in low-folate media WT cells became 68- to 310-fold more sensitive to the folate structural analogues, methotrexate, aminopterin and raltitrexed, with EC_50_ values ranging from 37 to 72 nM. The toxicity of the other inhibitors against WT cells was similar in both media. With DKO cells, none of the inhibitors showed any increase in sensitivity when cultured in low-folate media. Compared with WT, DKO cells are approximately fivefold less sensitive in high-folate medium to the DHFR inhibitors, aminopterin and methotrexate. Strikingly, the DKO parasites were ∼1000-fold less sensitive than WT to these same inhibitors in low-folate medium. Growth inhibition by the thymidylate synthase inhibitors, raltitrexed and pemetrexed, was completely abolished in DKO parasites, showing up to 11 000-fold resistance compared with WT. There was no marked change in sensitivity towards the lipophilic DHFR inhibitors pyrimethamine, trimetrexate or to 5-fluoro-oroate when compared across media or cell type. Likewise, the known PTR1 inhibitors belonging to the 2,4-diaminopteridine class, O/129 and triamterene, were also unchanged.

**Table 1:** Sensitivity of WT and DKO to DHFR–TS and PTR1 inhibitors.

	WT			DKO			Ratio DKO/WT	
			
Inhibitor	EC_50_ HMI9 (μM)	EC_50_ FDM (μM)	Ratio EC_50_	EC_50_ HMI9 (μM)	EC_50_ FDM (μM)	Ratio EC_50_	HMI9	FDM
Methotrexate	2.5 ± 0.2 (3)^a^	0.037 ± 0.007 (2)	68	17.9 ± 6.5 (2)	22.2 ± 5.2 (2)	0.8	7.2	484
Aminopterin	15.1 ± 2.3 (2)	0.049 ± 0.009 (3)	308	78.5 ± 5.9 (2)	75.1 ± 6.2 (2)	1.0	5.2	1 533
Raltitrexed	22.4 ± 2.1 (3)	0.072 ± 0.009 (3)	311	> 800 (2)	> 800 (2)	−	> 36	> 11 111
Pemetrexed	1.8 ± 0.1 (2)	1.9 ± 0.3 (2)	0.9	> 800 (2)	> 800 (2)	−	> 444	> 421
Pyrimethamine	17.2 ± 2.7 (3)	9.6 ± 1.3 (2)	1.8	35.6 ± 4.5 (2)	27.6 ± 2.6 (2)	1.3	2.1	2.9
Trimetrexate	1.2 ± 0.1 (3)	3.2 ± 0.3 (3)	0.4	4.1 ± 0.6 (3)	5.6 ± 0.8 (2)	0.7	1.9	1.8
5-Fluoro-orotic acid	2.8 ± 0.3 (3)	1.4 ± 0.3 (3)	1.6	4.2 ± 0.7 (2)	2.5 ± 0.5 (3)	1.7	1.9	1.7
O/129^b^	16.5 ± 1.7 (3)	7.4 ± 1.2 (2)	2.2	17.9 ± 2.1 (3)	9.7 ± 1.1 (3)	1.8	0.9	1.3
Triamterene	48.3 ± 5.1 (2)	45.2 ± 3.6 (2)	1.1	15.9 ± 1.1 (2)	58.9 ± 7.8 (2)	0.3	0.3	1.3

a *n* = number of independent experiments, where EC_50_ values are weighted means and standard errors of each independent determination.

b 2,4-Diamino-6,7-diisopropylpteridine.

### Can endogenous PTR1 compensate for lack of DHFR?

**Fig. 6:**
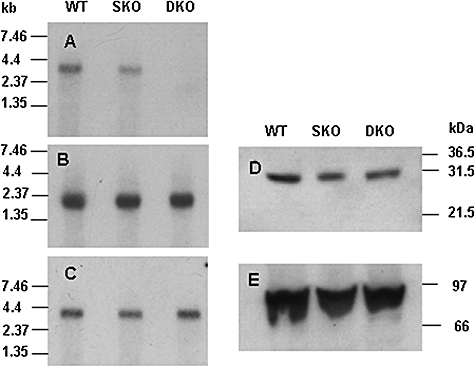
Northern and Western blot analysis of WT, SKO and DKO cells. Trypanosomes were grown for 24 h in HMI9 medium in the absence (WT and SKO) or presence of thymidine (DKO). A–C. RNA blots probed with *TbDHFR–TS*, *TbPTR1* and *TbINO1* respectively. RNA size markers are indicated to the left of the panels. D and E. Protein blots of parasite lysates probed with antiserum to PTR1 and BiP respectively. Approximately 20 μg of protein was loaded per lane. Standard size markers are indicated to the right of the panels.

PTR1 is known to reduce a broad spectrum of pterins and folates (Dawson *et al.*, 2006), raising the intriguing possibility that it may compensate for changes in DHFR activity in SKO and DKO lines. PTR1 levels in each cell line were analysed for RNA and protein content by Northern and Western blotting relative to suitable controls (Fig. 6). Densitometry of RNA blots indicated that the absolute ratio of *DHFR–TS* in WT, SKO and DKO is 1:0.5:0 (Fig. 6A). Using *INO1* [1-d-myo-inositol-3-phosphate synthase (Martin and Smith, 2005)] as a loading control (Fig. 6C), the corrected ratios are 1:0.6:0. *PTR1* transcripts are only marginally increased in SKO and DKO cell lines (1:1.1:1.1 respectively; Fig. 6B). After correction with *INO1*, the corresponding values are 1:1.4:1.1. Protein blots were probed with polyclonal antisera raised against *T. brucei* PTR1 (Fig. 6D) and the endoplasmic reticulum chaperone BiP (Fig. 6E). PTR1 levels are equal for all three cell lines, with expression levels relative to BiP being 1:0.9:0.9 for WT, SKO and DKO respectively.

**Fig. 7:**
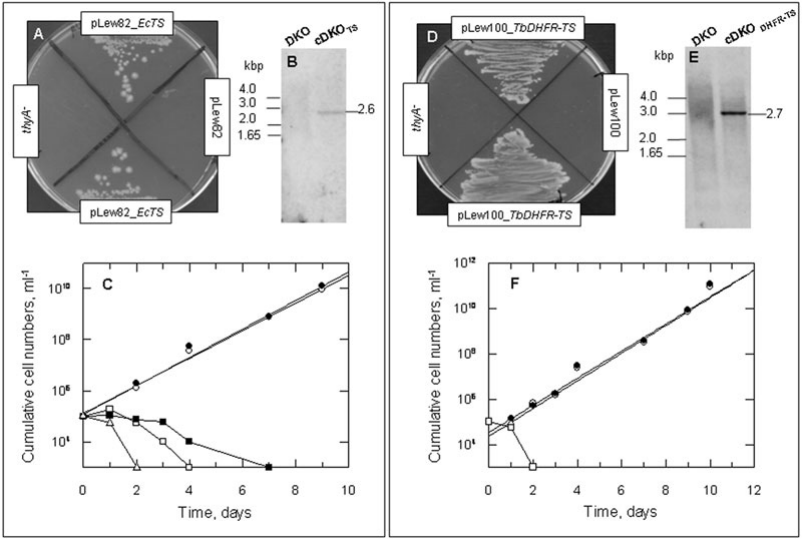
Complementation studies with *EcTS* and *TbDHFR–TS*. (A)–(C) Complementation with *E. coli TS* and (D)–(F) complementation with *T. brucei DHFR–TS*. A. Growth of *E. coli thyA*^-^ transformed with pLew82_*EcTS* or empty vector on thymidine-free medium. B. Southern analysis of *T. brucei* DKO transfected with pLew82_*EcTS* (cDKO_TS_). DNA was digested with SacI and SphI and probed with *EcTS* ORF. The size of the expected fragment is indicated on the right with standard markers on the left. C. Growth of cDKO_TS_ in HMI9 medium with or without thymidine. Circles, plus thymidine; squares, no thymidine present. Open symbols depict growth without tetracycline induction and closed symbols with tetracycline. Triangles, growth of DKO in the absence of thymidine. The lines represent the best fits to the equation describing exponential growth as described in *Experimental procedures*. D. Growth of *E. coli thyA*^-^ transformed with pLew100_*TbDHFR–TS* or empty vector on thymidine-free medium. E. Southern analysis of *T. brucei* DKO transfected with pLew100_*TbDHFR–TS* (cDKO_DHFR–TS_). DNA was digested with HindIII and StuI and probed with *TbDHFR–TS*. The size of the expected fragment is indicated on the right with standard markers on the left. F. Growth of cDKO_DHFR–TS_ in HMI9 medium with and without thymidine (open and closed circles respectively). Open squares, growth of DKO in the absence of thymidine. The lines represent the best fits to the equation describing exponential growth as described in *Experimental procedures*. Symbols on the *x*-axis indicate that cell densities are below the limits of detection (i.e. < 10^3^ ml^−1^).

Although PTR1 levels are unchanged in the knockout lines, it is possible that endogenous activity is nonetheless sufficient to compensate for the loss of DHFR activity. To determine whether the thymidylate cycle could be sustained by endogenous PTR1, we constructed a recovery plasmid containing the thymidylate synthase gene from *Escherichia coli*. This plasmid (pLew82*_EcTS*) was able to complement *E. coli thyA*^-^ cells grown on thymidine-deficient medium indicating that TS was functional (Fig. 7A). This expression construct was transfected into DKO cells and lines selected for phleomycin resistance in HMI9 medium. Integration of the *EcTS* in the conditional DKO (cDKO_TS_) cells at the correct locus was confirmed by Southern blot analysis using the *EcTS* as a probe (Fig. 7B). Growth was monitored in HMI9 (with and without thymidine and/or tetracycline) to determine whether the addition of TS alone could maintain the thymidylate cycle in the absence of DHFR (Fig. 7C). The TS add-back grew normally in HMI9 containing thymidine (generation times 13 and 13.4 h, respectively, with or without tetracycline), but, in its absence, no viable trypanosomes were visible beyond day 4. Nonetheless, when compared with the DKO grown in the absence of thymidine, survival was prolonged by 2 and 3 days, respectively, in the absence and presence of tetracycline.

**Fig. 8:**
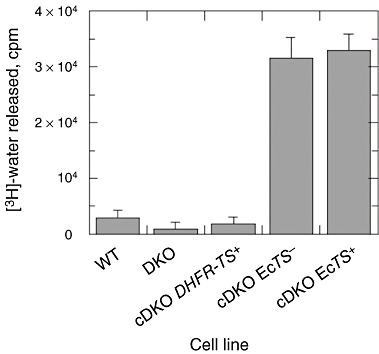
TS activity in WT and transgenic *T. brucei*. TS activity was determined by release of [^3^H]-water from [^3^H]-dUMP as described in *Experimental procedures*. Error bars represent the standard error of triplicate assays. The minus and plus signs refer to growth in HMI9 medium in the absence or presence of tetracycline respectively.

To demonstrate that the *E. coli* TS was also functional in *T. brucei*, extracts were prepared from cell lines cultured in the presence of thymidine and assayed radiometrically for enzyme activity (Fig. 8). Irrespective of tetracycline induction, cDKO_TS_ cells showed ∼10-fold greater enzymatic activity than WT cells (2838 ± 1438 cpm) confirming that functional TS is present in this transgenic line. The DKO line showed activity that was not significantly different from zero (862 ± 1283 cpm), confirming the absence of TS activity as demonstrated by thymidine auxotrophy (Figs 3 and 4).

To demonstrate that deletion of *DHFR–TS* had not resulted in other genomic mutations, a copy of *TbDHFR–TS* (with two internal HindIII restriction sites ablated) was cloned into pLew100 (Wirtz *et al.*, 1999). The resulting construct was able to rescue *thyA*^-^*E. coli* (Fig. 7D) indicating that the modified *TbDHFR–TS* was functional. pLew100_*TbDHFR–TS* was then transfected into the DKO null and found to be correctly targeted to the ribosomal DNA locus resulting in cDKO_DHFR–TS_ cells (Fig. 7E). cDKO_DHFR–TS_ cells were able to grow in the absence of thymidine, with or without induction by tetracycline (generation times for both are 12 h; Fig. 7F). Enzymatic activity approaching WT levels was found in this cell line (Fig. 8), confirming that TS was functional. Loss of tetracycline control is a frequently observed phenomenon with the pLew family of vectors (Krieger *et al.*, 2000; Milne *et al.*, 2001; Chang *et al.*, 2002; Martin and Smith, 2006a). Loss of expression of the TETR protein or mutation or ablation of the tet operator site has been proposed as possible mechanisms of escape from tetracycline repression. However, only one study has demonstrated that this results from deletion of the *TETR* gene (Roper *et al.*, 2002). This is not the case here, as PCR confirmed the presence of *TETR* (not shown). Thus, loss of tetracycline control in our experiments must be due to another mechanism.

### Virulence in mice

**Fig. 9:**
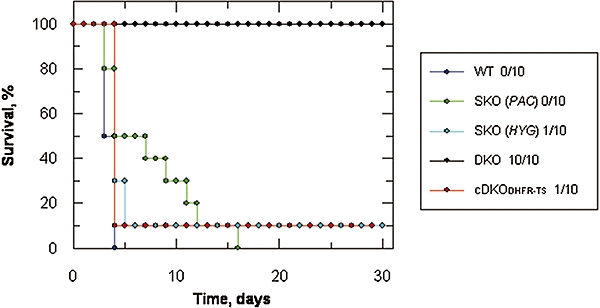
Virulence of WT, SKO and DKO *T. brucei* infections in mice. Each data set represents the combined results of two independent experiments presented as a Kaplan–Meier survival plot. The figures in the legend are the numbers of survivors per infected group. As the cDKO_DHFR–TS_ was not responsive to tetracycline induction these data sets have been combined. Symbols: WT, dark blue; SKO (PAC), green; SKO (HYG), light blue; DKO, black; cDKO, red.

The nutritional environment *in vivo* is considerably different from *in vitro* culture conditions underlining the need for target validation studies to be carried out in animal models, where possible (Frearson *et al.*, 2007). To confirm the essentiality of DHFR–TS, mice were inoculated with WT or genetically modified parasites and the course of the infection monitored over a 30-day period. A survival curve for the various infections is shown in Fig. 9. Mice infected with WT cells were unable to survive beyond 3–4 days, whereas DKO-infected mice remained completely free of parasites and survived beyond 30 days. This indicates that the thymidine level in mouse plasma (1 μM; Clarke *et al.*, 2000) is not sufficient to sustain DKO cell viability *in vivo*, consistent with the *in vitro* data presented in Fig. 4. Virulence was restored in cDKO_DHFR–TS_ cells indicating that the selectable markers *PAC* and *HYG* had not suppressed the virulence phenotype. SKO infections were also lethal. In one experiment with SKO (*PAC*), survival times were extended to up to 16 days, but this was not reproduced in a second experiment. Virulence of a second single-knockout line (SKO *HYG*) was also similar to WT, except for one mouse that remained aparasitaemic throughout the 30-day experiment. These results demonstrate definitively that DHFR–TS is absolutely essential for parasite survival in the host. They also suggest that sustained inhibition of DHFR–TS by >50% would be required for a chemotherapeutic effect.

## Discussion

The genetic studies presented here clearly indicate that DHFR–TS is essential for growth and survival of the African trypanosome *in vitro* and *in vivo.* Nutritional rescue of the *DHFR–TS* null is specific for thymidine and cannot be replaced by other pyrimidines, consistent with a specific requirement of both enzymes in the thymidylate cycle (Fig. 1). The finding that PTR1 levels are unchanged in DKO cells and the fact that genetic rescue can be achieved by ectopic expression of *TbDHFR–TS*, but not with functionally active *EcTS*, suggests that PTR1 is unable to compensate for complete loss of DHFR activity. PTR1 from *T. brucei* is fourfold less efficient in reducing dihydrofolate than the *L. major* enzyme and is less sensitive to inhibition by methotrexate (Dawson *et al.*, 2006). However, it is pertinent to note that *DHFR* mutants in *E. coli* are always associated with compensatory mutations in *ThyA* to maintain the delicate balance between formation and removal of dihydrofolate by TS and DHFR respectively (Ahrweiler and Frieden, 1988; Howell *et al.*, 1988). Thus, the failure to restore viability with *EcTS* could be due to an imbalance between PTR1 and TS activity, which is 10-fold higher than in WT cells. Moreover, although DHFR in *T. brucei* may be a drug target *per se* without recourse to inhibiting PTR1, amplification of PTR1 could represent a potential resistance mechanism to DHFR inhibitors, as observed in *Leishmania* spp. (Bello *et al.*, 1994; Nare *et al.*, 1997b; Ouellette *et al.*, 2002). However, unlike *Leishmania* spp., gene amplification in African trypanosomes is extremely rare. To our knowledge, amplification of the inosine monophosphate dehydrogenase gene in *Trypanosoma gambiense* in response to selection by mycophenolic acid is the only example reported in the literature (Wilson *et al.*, 1994).

The fact that *T. brucei* DHFR–TS null mutants are non-viable *in vivo* is in contrast to *L. major* DHFR–TS null mutants, which cause persistent avirulent infections in mice. Presumably, this is due to sufficient amounts of thymidine being available in the parasitophorous vacuole of the host macrophage (Titus *et al.*, 1995). This interesting phenomenon of persistence has been exploited as a potential live vaccination strategy in leishmaniasis (Veras *et al.*, 1999). In complete contrast, *T. brucei* DKO lines are totally incapable of establishing or maintaining an infection in mice. Given the narrow window of thymidine concentrations that can support growth of *T. brucei in vitro*, this is likely to be a consequence of the extremely low concentrations of thymidine in plasma: ∼1 μM in mice or ∼0.1 μM in humans (Clarke *et al.*, 2000).

The underlying mechanisms involved in ‘thymine-less death’ in prokaryotes and eukaryotes are not fully understood (Ahmad *et al.*, 1998). The primary event following inhibition of TS activity is extreme depletion of the dTTP pool with a concomitant increase in dUMP and its subsequent phosphorylation to form dUTP (Van Triest *et al.*, 2000; Ackland *et al.*, 2003; Gmeiner, 2005). If the capacity of dUTPase to hydrolyse dUTP is exceeded, then a futile cycle of misincorporation of uracil into DNA, followed by base excision by uracil-DNA glycosylase, takes place, ultimately causing irreparable chromosome damage and cell death (Ahmad *et al.*, 1998; Van Triest *et al.*, 2000; Tinkelenberg *et al.*, 2002). However, we found no evidence for death by an apoptotic-like mechanism as frequently observed in higher eukaryotes (Van Triest *et al.*, 2000). Rather, the morphological phenotype of an enlarged flagellar pocket observed here resembles the ‘big-eye’ phenotype reported for clathrin knock-down by RNAi in *T. brucei* (Allen *et al.*, 2003). It is not immediately apparent what the common mechanism might be, if any. Further studies on the biochemical processes leading to cell death due to thymidine depletion are required.

The inhibitors used in this study have uncovered some interesting features of folate metabolism in *T. brucei* with potential implications for target validation and drug discovery. Phenotypic drug screening against these parasites using standard HMI9 medium is not ideal for inhibitors targeting folate and thymidylate metabolism. For example, high folate levels markedly attenuate the cytotoxic effect of antifolate inhibitors such as methotrexate and aminopterin (both DHFR inhibitors) and raltitrexed (a TS inhibitor) that are structurally similar to physiological folates. The precise mechanism of attenuation remains to be determined, but the high folate in HMI9 medium could competitively inhibit drug uptake into cells, interfere with cellular retention by competition for polyglutamylation by folylpolyglutamyl synthetase or compete for the active sites of target enzymes as reported for mammalian systems (Kane *et al.*, 1986; Jansen *et al.*, 1989; 1998; Barnes *et al.*, 1999; Backus *et al.*, 2000; van der Wilt *et al.*, 2001). Interestingly, the trypanocidal potency of the TS inhibitor, pemetrexed (Wang *et al.*, 2003), is not affected by high folate levels in the medium, despite a pronounced (440-fold) decrease in potency against the DKO cell line. This may be due to a different mode of uptake or reduced polyglutamylation compared with the other folate analogues. Further transport and metabolism studies are required to test these possibilities.

Deletion of DHFR–TS causes a huge loss in inhibitor potency (>500-fold) in cells cultured in low-folate medium, indicating that these enzyme activities are the primary targets for methotrexate, aminopterin, raltitrexed and pemetrexed. However, this effect is not observed with lipophilic inhibitors such as pyrimethamine, a potent inhibitor of *Tb*DHFR (Gamarro *et al.*, 1995), or trimetrexate, a potent inhibitor of *Trypanosoma cruzi* DHFR (
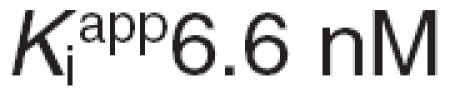
) which has an EC_50_ of 26 nM against *T. cruzi* amastigotes (Senkovich *et al.*, 2005). Neither is it observed for 5-fluoro-orotate, a prodrug of 5-FdUMP. The reason for this is not known. However, the high concentrations (>1 μM) required to inhibit growth point to factors such as poor cellular pharmacokinetics (absorption, distribution, metabolism and excretion) or poor potency against the target with other ‘off-target effects’ predominating in cell killing. The latter explanation is likely in the case of 5-fluo-orotate, as fluorinated pyrimidine analogues are known to have pleiotropic effects such as misincorporation into RNA and DNA in other cells (Van Triest *et al.*, 2000). Interestingly, the sensitivity of *T. brucei* to raltitrexed and 5-fluoro-orotate is the inverse of what is observed for malaria parasites, where raltitrexed (ICI D1694) is virtually inactive (EC_50_ >20 μM) (Rathod and Reshmi, 1994), yet 5-fluoro-orotate is highly active (EC_50_ 6 nM) (Rathod *et al.*, 1989).

In conclusion, we have evaluated DHFR–TS as a drug target in African trypanosomes and obtained definitive genetic evidence and compelling chemical evidence suggesting that these enzymes are sufficiently validated to warrant entry into a drug discovery pipeline. Enzymatic and structural studies of this enzyme are underway to facilitate this process.

## Experimental procedures

### Trypanosome culture

*Trypanosoma brucei* bloodstream-form ‘single marker’ S427 (*T7RPOL TETR NEO*) and knockouts were cultured in HMI9 medium (Hirumi and Hirumi, 1989) supplemented with 2.5 μg ml^−1^ G418 to maintain expression of T7 RNA polymerase and the tetracycline repressor protein (Wirtz *et al.*, 1999). HMI9 medium contains high concentrations of folate principally from the Iscove's modified Dulbecco's medium (IMDM) and the 10% (v/v) Serum Plus components (SAFC Biosciences). Serum Plus is a proprietary medium supplement containing 20% fetal bovine serum (FBS), growth-promoting factors, hormones, lipids and 2.72 mg l^−1^ folic acid (6.2 μM folate, excluding FBS; personal communication from SAFC Biosciences). HMI9 therefore contains 7.9 μM folate, not including folate from FBS (12% v/v, final). Folate free IMDM is not commercially available, so a FDM equivalent to HMI9 was prepared using folate-free RPMI 1640 supplemented with 50× MEM amino acid solution (Invitrogen) to provide amino acid and other nutrient concentrations equivalent to those found in HMI9 (see Table S1). Other components were the same as HMI9 (14 mM glucose, 25 mM HEPES, 2 mM pyruvate, 0.5 mM bathocuproine disulphonic acid, 1.5 mM cysteine, 0.2 mM 2-mercaptoethanol, 1 mM hypoxanthine, 0.16 mM thymidine and 10% FBS) except Serum Plus was reduced to 0.5% so that the total FBS was 10.1% (see Table S1). Excluding the contribution from the serum components, FDM contains 31 nM folate.

Cultures were initiated with 1 × 10^5^ cells ml^−1^ and subcultured when cell densities approached 1–2 (× 10^6^) ml^−1^. The single marker WT, SKO and DKO cell lines were also grown continuously in the presence of the appropriate drug selection (see below for further details). Once established, DKO cell lines were also grown in the absence of additional drug selection. Generation times (*g*) were determined by non-linear regression using GraFit with proportional weighting fitted to the following equation:

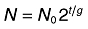

where *N*_0_ is the starting cell density at zero time and *N* is cell density at time, *t*.

### Generation of knockout and recovery constructs

Primers used in this study (Table S2) are based on the *T. brucei DHFR–TS* GeneDB sequence (Tb927.7.5480, 927 strain) and *E. coli ThyA* (Accession No. J01710, K-12, MG1655 strain). All constructs were sequenced to confirm correct assembly. To generate the *T. brucei* gene replacement cassettes, the region containing the 303 bp of the 5′-UTR to the 286 bp 3′-UTR flanking the open reading frame (ORF) of *T. brucei DHFR–TS* was amplified from genomic DNA, using *Pfu* polymerase. This was used as a template for the amplification of the individual regions for the assembly of the replacement cassettes containing the selectable drug resistance genes puromycin *N*-acetyl transferase (*PAC*) and hygromycin phosphotransferase (*HYG*), as previously described (Martin and Smith, 2006b). To generate a recovery construct pLew100_*TbDHFR–TS*, the *DHFR–TS* ORF was amplified using *TbDHFR–TS*-XhoI fwd and *TbDHFR–TS*-BamHI rev primers, and cloned into pCR-Blunt II TOPO (Invitrogen). This was used to introduce silent mutations to remove two HindIII sites within the gene at Leucine 236 (L236) and Alanine 452 (A452) sequentially with the following sets of primers HindIII-L236 fwd, HindIII-L236 rev and HindIII-A452 fwd HindIII-A452 rev respectively (Table S2). This TOPO clone was used as a template to generate a PCR product containing 5′-HindIII and 3′-PacI restriction sites which was subcloned into a modified pLew100 vector containing blasticidin S transferase (*BSD*) replacing *NEO* as the selectable gene marker (kindly supplied by Dr Kirstee Martin).

The recovery construct, pLew82_*EcTS* containing the *E. coli* gene for thymidylate synthase (*ThyA*), was engineered as follows. *ThyA* (Accession No. J01710) was amplified from *E. coli* (K-12, MG1655 strain) gDNA using *EcThyA*-HindIII fwd and *EcThyA*-BamHI rev primers (Table S2) and the product cloned into pCR-Blunt II TOPO (TOPO_*EcTS*). The ORF was subcloned into pLew82 containing the resistance marker gene bleomycin.

### Generation of an *E. coli thyA*^-^ cell line and functional complementation of TS recovery constructs

An *E. coli* thymidylate synthase deficient (*thyA*^-^) cell line derived from *E. coli* BL21 (DE3) Star cells (Invitrogen) was engineered using the TargeTron™ Gene Knockout System (Sigma-Aldrich) as described in the manufacturer's instructions. Briefly, the TOPO_*EcTS* clone was used as the template for the intron retargeting PCR using primers IBS-*EcThyA* and EBS2-*EcThyA* (Table S2) that were chosen at Sigma's TargeTron Design Site (http://www.sigma-genosys.com/targetron/). The disrupted *thyA*^-^ cells were selected using kanamycin and *ThyA*-specific disruption was verified by colony-PCR screening using *EcThyA*-HindIII fwd and EBS2-*EcThyA* primers. The resulting *thyA*^-^*E. coli* was transformed with the pLew82_*EcTS* or pLew100_*TbDHFR–TS* recovery constructs and plated out onto LB containing 50 μg ml^−1^ carbenicillin and 1 μg ml^−1^ tetracycline to confirm that these plasmids contained a functional thymidylate synthase gene. Negative controls of the empty vector and cells alone were included.

### Generation of bloodstream-form *T. brucei* knockout lines

Knockout and recovery constructs were prepared using QIAprep Miniprep Plasmid Kit (Qiagen). DNA was digested with NotI, ethanol precipitated and redissolved in sterile water at a final concentration of 1 μg μl^−1^. Trypanosomes were electroporated as described previously (Wirtz *et al.*, 1999; Chang *et al.*, 2002; Roper *et al.*, 2002; Martin and Smith, 2006b). Transformed SKO cells containing the *PAC* or *HYG* genes were cultured in the presence of 0.1 μg ml^−1^ puromycin or 4.0 μg ml^−1^ hygromycin respectively. The SKO *PAC* line was used to produce a DKO line using the *HYG* knockout construct to replace the second endogenous copy of *DHFR–TS* by selection with hygromycin and puromycin. Transfection of the pLew82_*EcTS* and pLew100_*TbDHFR–TS* recovery constructs into the DKO cells resulted in the selection of conditional DKO (cDKO) cells with 2.5 μg ml^−1^ phleomycin and blasticidin respectively.

### DNA and RNA analysis of KO cells

Confirmation of the correct integration of the knockout and recovery constructs was undertaken with restriction digests of 5 μg of gDNA with subsequent Southern blot analysis as described previously (Martin and Smith, 2006b) using DNA probes specific for *DHFR–TS*, *PAC*, *HYG* and *ThyA*. RNA was prepared from approximately 2 × 10^8^ cells (WT and SKO cells grown in HMI9 lacking thymidine and DKO cells in HMI9) using Qiagen RNeasy Mini Kit. The RNA samples (5 μg) were denatured in formaldehyde/formamide premix at 65°C for 15 min and run on a 1.2% agarose/2% formaldehyde/1× MOPS gel and transferred to Hybond-N nylon membrane (Amersham Biosciences). Blots were hybridized sequentially with [α-^32^P]-dCTP-labelled probes for *TbPTR1*, *TbDHFR–TS* and *TbINO1* prepared with Rediprime (Amersham Biosciences).

### Western blot analysis of cell lysates

Polyclonal antisera against *T. brucei* PTR1 were raised in adult male Wistar rats. An initial injection of 100 μg of purified antigen (Dawson *et al.*, 2006), emulsified in complete Freund's adjuvant, was followed by two identical booster injections of antigen emulsified in Freund's incomplete adjuvant at 2-week intervals.

Wild-type and SKO trypanosomes were grown in thymidine-deficient HMI9 media while DKO cells were grown in HMI9 supplemented with thymidine. Mid-log cells (∼1 × 10^9^ cells) were harvested by centrifugation (800 *g*, 10 min, 25°C), aspirated and pellets re-suspended in 1 ml of ice-cold PBS (137 mM NaCl, 8 mM KCl, 10 mM Na_2_HPO_4_, 2 mM KH_2_PO_4_, pH 7.4) containing a cocktail of protease inhibitors (Calbiochem, Protease Inhibitor Cocktail Set III). Following centrifugation (5000 *g*, 2 min, 25°C), cell pellets were further re-suspended in 500 μl of ice-cold PBS containing protease inhibitors, 1 mM EDTA and 1 μg ml^−1^ of both DNase and RNase. For biological safety, samples were freeze-thawed five times prior to mechanical disruption with sterile glass beads (Sigma, bore size 425–600 μm) in a micro pestle. Lysates were centrifuged (13 000 *g*, 20 min, 4°C), the resulting supernatants were collected and protein concentration was determined.

Cell extracts (20 μg) were then separated on SDS-PAGE and subsequently transferred onto nitrocellulose. After blocking with 5% skimmed milk in PBS at room temperature for 1 h, the blot was incubated with *T. brucei* PTR1 polyclonal antiserum (1/1000 dilution) or *T. brucei* BiP (kindly supplied by Dr M.L. Güther, originally from Dr J. Bangs) at room temperature for 1 h, washed in PBS containing 0.1% (v/v) Tween 20 and then incubated with a secondary rabbit anti-rat (IgG) antibody (Dako; 1/10 000 dilution). The immunoblots were subsequently developed using the ECL® plus (enhanced chemiluminescence) system from Amersham Biosciences.

### Thymidylate synthase assays

Thymidylate synthase activity in cell lysates was carried out as described (Nirmalan *et al.*, 2004) with minor modifications. Approximately 2 × 10^8^ cells were prepared from WT, DKO, cDKO_DHFR–TS_ (with tetracycline induction), cDKO_TS_ (with and without tetracycline induction), osmotically lysed with ice-cold distilled water, frozen and thawed three times in liquid nitrogen and finally re-suspended in an equal volume of 2× TS assay buffer (0.4 M Tris-HCl pH 7.4 containing 2 mM EDTA, 40 mM 2-mercaptoethanol, 200 mM NaF and 30 mM CMP). The suspension was centrifuged for 10 min, at 12 000 *g* at 4°C and the resulting supernatant was used to determine the TS activity. This involves a tritium release assay using [5-^3^H]-dUMP (Moravek Biochemicals) and (*6R,S*)*-5,10*-methylene-*5,6,7,8*-tetrahydrofolic acid (Schircks Laboratories). In brief the standard assay consisted of 200 μl of cell lysate prepared in TS assay buffer with the addition of 5 μl of 6.5 mM methylene tetrahydrofolate and 5 μl of [5-^3^H]-dUMP (19 Ci mmol^−1^). Assay mixtures were incubated at 37°C for 1 h and reactions stopped with the addition of 50 μl of ice-cold 100% (w/v) TCA. After incubation for 10 min on ice, residual [5-^3^H]-dUMP was removed by five extractions with activated charcoal suspension. After the final extraction step, 200 μl of aqueous supernatant was counted in 3 ml of scintillation fluid. Samples were analysed in triplicate and background counts subtracted from negative control reaction mixtures containing heat-inactivated lysate.

### EC_50_ determination of inhibitors against WT and DKO cells

Antifolates were tested against WT and DKO cells in HMI9 and FDM (both containing 160 μM thymidine). Serial doubling dilutions of drugs (10–20 dilutions from 10 mM stocks prepared in 10% DMSO) were added to flat-bottomed 96-well plates containing 1 × 10^4^ cells ml^−1^ in a final volume of 0.2 ml in either HMI9 or FDM. Plates were incubated for 72 h at 37°C/5% CO_2_ and cell densities were determined using Alamar Blue (Raz *et al.*, 1997). EC_50_ values were calculated using GraFit software (Erithacus Software) with a non-linear 4-parameter robust curve fit in triplicate. All inhibitors were from Sigma Chemical, except pemetrexed (International Laboratory USA), trimetrexate (Tocris) and raltitrexed (Tomudex, ZD1694, from Astra Zeneca).

### FACS analysis

Wild-type and DKO cells were grown to 1 × 10^6^ cells ml^−1^ in HMI9 medium, centrifuged (2500 *g*, 10 min, 25°C) and washed twice in HMI9 medium lacking thymidine. The cell pellets were re-suspended in thymidine-deficient medium and incubated for a further 24 h. Cells were collected by centrifugation and washed twice in PBS before preparation for FACS analysis (Tu and Wang, 2005). Cells were finally adjusted to 5 × 10^5^ cells ml^−1^, re-suspended in 500 μl of PBS containing 50 μg ml^−1^ propidium iodide, 50 μg ml^−1^ RNase and 0.1% Triton X-100, incubated at room temperature for 20 min in the dark and analysed using FACSort analytical flow cytometer using Cellquest software (BD Biosciences).

### Morphological analysis

Cells were examined following fixation with methanol or 4% (v/v) paraformaldehyde and stained with Giemsa or 4′,6-diamidino-2-phenylindole (DAPI), respectively, using a Carl Zeiss light and fluorescent microscope. For electron microscopy, WT and DKO cells (grown in the presence and absence of thymidine) were fixed for 24 h in 2% (v/v) glutaraldehyde and 4% (v/v) paraformaldehyde in 0.2 M PIPES, pH 7.2. For transmission electron microscopy cells were prepared as described previously (Broadhead *et al.*, 2006), post-fixed for 1 h at 4°C with 1% (v/v) osmium tetroxide in 100 mM phosphate buffer (pH 6.5) and rinsed briefly in water prior to fixing or staining *en bloc* with 3% (v/v) aqueous uranyl acetate. Cells were rinsed further in distilled water and subsequently dehydrated through a graded ethanol series with a final wash in propylene oxide, prior to embedding in Durcupan resin. Sections were stained with 3% (v/v) aqueous uranyl acetate and Reynold's lead citrate prior to examination using a JEOL-1200 EX TEM. For scanning electron microscopy, the same fixation and *en block* staining protocol was applied. After ethanol dehydration series the samples were critical-point dried and the membranes coated with gold-palladium by sputter staining and examined using a Philips XL 30 SEM.

### *In vivo* studies

All cell lines were cultured for 24 h in the absence of selectable drugs before mice (five per group) were infected with a single intraperitoneal injection of 10^5^ parasites in 0.2 ml of glucose saline. One set of animals were administered with 2.5 μg ml^−1^ doxycycline in their drinking water 3 days prior to infection with the cDKO_DHFR–TS_ and freshly prepared every second day for the duration of the experiment. Animals were inspected daily for clinical signs of infection (reduced activity, ruffled fur, increased respiration and prostration or collapse) and wet smears of tail blood were examined microscopically (×40 lens and ×10 objective) in 20 high-power fields for parasites. When more than five parasites were visible per high-power field 1 μl of tail blood was drawn by capillary action into a Microcap glass capillary (Sigma), diluted 200-fold with glucose saline and parasite density was determined microscopically using a Neubauer haemocytometer. Any animal with a parasitaemia exceeding 1 × 10^8^ ml^−1^ was humanely killed as pilot experiments had established that animals were unable to survive a further 24 h with WT (SM) strain.

## Supplementary material

This material is available as part of the online article from: http://www.blackwell-synergy.com/doi/abs/10.1111/

j.1365-2958.2008.06305.x

(This link will take you to the article abstract).
