# Supplementary material for: Chemical and genetic validation of dihydrofolate reductase–thymidylate synthase as a drug target in African trypanosomes
Source: Mol Microbiol. 2008 Jun 16;69(2):520–33. doi: 10.1111/j.1365-2958.2008.06305.x (PMC2610392; doi:10.1111/j.1365-2958.2008.06305.x)
Supplement: Supplementary file 1 [file mmi0069-0520-SD1.doc]

**Table S1 Composition of HMI9 and Folate Deficient Medium (FDM)**

| **COMPONENTS** | **HMI9 (mM)** | **FDM (mM)** | **Ratio**  **HMI-9 / FDM** |
| --- | --- | --- | --- |
| **Amino acids** | | | |
| Hydroxy L-proline | 0 | 0.153 | 0.00 |
| Glycine | 0.400 | 0.133 | 3.01 |
| L-Alanine | 0.281 | 0 | ∞ |
| L-Arginine | 0.398 | 1.75 | 0.23 |
| L-Asparagine | 0.167 | 0.379 | 0.44 |
| L-Aspartic acid | 0.226 | 0.150 | 1.51 |
| L-Cystine 2HCl | 0.381 | 0.285 | 1.34 |
| L-Glutamic Acid | 0.510 | 0.136 | 3.75 |
| L-Glutamine | 4.00 | 2.05 | 1.95 |
| L-Histidine | 0.200 | 0.297 | 0.67 |
| L-Isoleucine | 0.802 | 0.782 | 1.03 |
| L-Leucine | 0.802 | 0.782 | 1.03 |
| L-Lysine hydrochloride | 0.798 | 0.670 | 1.19 |
| L-Methionine | 0.201 | 0.202 | 1.00 |
| L-Phenylalanine | 0.400 | 0.291 | 1.37 |
| L-Proline | 0.348 | 1.82 | 0.19 |
| L-Serine | 0.400 | 0.286 | 1.40 |
| L-Threonine | 0.798 | 0.568 | 1.40 |
| L-Tryptophan | 0.0784 | 0.745 | 0.11 |
| L-Tyrosine disodium salt | 0.462 | 0.248 | 1.86 |
| L-Valine | 0.803 | 0.571 | 1.41 |
| **Vitamins** | | | |
| Biotin | 0.0000533 | 0.000820 | 0.07 |
| Choline chloride | 0.0286 | 0.0214 | 1.34 |
| D-Calcium pantothenate | 0.00839 | 0.000524 | 16.01 |
| Folic Acid | 0.00807^a^ | 0.0000309^b^ | 261.17 |
| i-Inositol | 0.0400 | 0.194 | 0.21 |
| Niacinamide | 0.0328 | 0.00820 | 4.00 |
| Pyridoxal / pyridoxine | 0.0196^c^ | 0.00485^d^ | 4.04 |
| Para-Aminobenzoic Acid | 0 | 0.00730 | 0 |
| Riboflavin | 0.00106 | 0.000532 | 1.99 |
| Thiamine hydrochloride | 0.0119 | 0.00297 | 4.01 |
| Vitamin B12 | 0.0000096 | 0.0000037 | 2.59 |
| **Salts** | | | |
| Calcium ions | 1.49^e^ | 0.424^f^ | 3.51 |
| Nitrate ions | 0.000752^g^ | 0.8480^h^ | 0.0009 |
| Magnesium Sulphate (MgSO_4_) | 0.814 | 0.407 | 2.00 |
| Potassium Chloride (KCl) | 4.40 | 5.33 | 0.83 |
| Sodium Bicarbonate (NaHCO_3_) | 36.00 | 23.81 | 1.51 |
| Sodium Chloride (NaCl) | 77.59 | 103.45 | 0.75 |
| Sodium Phosphate | 0.906 | 5.64 | 0.16 |
| Sodium Selenite (Na_2_SeO_3_.5H_2_0) | 0.0000658 | 0 | ∞ |
| **Other components** | | | |
| D-Glucose (Dextrose) | 25.00 | 11.2 (25.1) ^i^ | 1.0 |
| HEPES | 25.03 | 25 | 1.0 |
| Phenol Red | 0.0399 | 0.0133 | 0.33 |
| Sodium Pyruvate (total) | 1.0 (2.0) ^i^ | 0 (2.0) ^i^ | 1.0 |
| Glutathione (reduced) | 0 | 0.00326 | 0 |
| **Additional Supplements** |  |  |  |
| D-Glucose | 0 | 14.0 (see above) | - |
| HEPES | 0 | 25.0 (see above) | - |
| Bathocuproine sulphonate | 0.05 | 0.05 | 1.0 |
| Cysteine | 1.5 | 1.5 | 1.0 |
| Hypoxanthine | 1.0 | 1.0 | 1.0 |
| Mercaptoethanol | 0.2 | 0.2 | 1.0 |
| Pyruvate | 1.0 (see above) | 2.0 (see above) | - |
| Thymidine | 0.16 | 0.16 | 1.0 |
| Foetal bovine serum (%) | 10 | 10 | 1.0 |
| Serum Plus (%) | 10 | 0.5 | 20 |

**Notes:**

a) From Iscove’s Modified Dulbecco’s Media and 10% Serum Plus, excluding any contribution from FBS

b) From 0.5% Serum Plus and folate free RPMI medium, excluding any contribution from FBS

c) As pyridoxal hydrochloride

d) As pyridoxine hydrochloride

e) As CaCl_2_

f) As Ca(NO_3_)_2_

g) As KNO_3_

h) As Ca(NO_3_)_2_

i) Values in parentheses include additional supplements

**Table S2. Primers used for construction of knockout and rescue constructs.** Upper case letters refer to nucleotides corresponding to gene sequences in *T. brucei* or in *E. coli*; lower case refers to additional sequences used in generating constructs. Restriction endonuclease sites are underlined and silent mutation sites used to ablate internal HindIII sites in *TbDHFR-TS* are indicated in bold and underlined.

| **Primer name** | **Primer sequence** |
| --- | --- |
| 5´UTR-NotI _fwd  5´UTR-HindIII/PmeI rev  3´UTR-PmeI/BamHI fwd  3´UTR-NotI rev  *TbDHFR-TS*-XhoI fwd  *TbDHFR-TS*-BamHI rev  HindIII-L236 fwd  HindIII- L236 rev  HindIII-A452 fwd  HindIII-A452 rev  *TbDHFR-TS*-HindIII fwd  *TbDHFR-TS*-PacI rev  *EcThyA-*HindIII fwd  *EcThyA-*BamHI rev  IBS-*EcThyA*  EBS1d-*EcThyA*  EBS2-*EcThyA* | 5´-ataagaatgcggccgcgtctgctcaaatcgtcg-3´  5´-gtttaaacttacggaccgtcaagcttgaccccctttggtattgc-3´  5´-gacggtccgtaagtttaaacggatccgaatgggtggaaatgc-3´  5´-ataagtaagcggccgccaataatgacaaaggaac-3´  5´-ctcgagatgctcagtcttacgcgt-3´  5´-ggatccctacaccgccatctccatag-3´  5´-CAGTTTGAGAAGCT**C**GTCCCCCGAAACAGC-3´  5´-GCTGTTTCGGGGGAC**G**AGCTTCTCAAACTG-3´  5´-CCTCATGGCAAAAGC**C**TCTGGACTTCGGCC-3´  5´-GGCCGAAGTCCAGA**G**GCTTTTGCCATGAGG-3´  5´-aagcttatgctcagtcttacgcgt-3´  5´-ttaattaactacaccgccatctccatag-3´  5´-aagcttatgaaacagtatttagaactg-3´  5´-ggatccttagatagccaccggcgctttaatg-3´  5´-AAAAAGCTTATAATTATCCTTAACGCACGTGGCAG  TGCGCCCAGATAGGGTG-3´  5´-CAGATTGTACAAATGTGGTGATAACAGATAAGTCG  TGGCAACTAACTTACCTTTCTTTGT-3´  5´-TGAACGCAAGTTTCTAATTTCGATTTGCGTTCGATA  GAGGAAAGTGTCT-3´ |
